# Creating artificial Rhino Horns from Horse Hair

**DOI:** 10.1038/s41598-019-52527-5

**Published:** 2019-11-08

**Authors:** Ruixin Mi, Z. Z. Shao, F. Vollrath

**Affiliations:** 1 Department of Zoology, University of Oxford, OX1 3SZ Oxford, UK; 2 State Key Laboratory of Molecular Engineering of Polymers, Advanced Materials Laboratory, Fudan University, Shanghai, 200433 China

**Keywords:** Biological models, Biopolymers in vivo

## Abstract

Demand for rhino horn is driving poaching with devastating effect for the few individuals left of the few species surviving from this once numerous, widespread and cosmopolitan clade of pachyderms. We bundled together tail hairs of the rhino’s ubiquitous near relative, the horse, to be glued together with a bespoke matrix of regenerated silk mimicking the collagenous component of the real horn. This approach allowed us to fabricate composite structures that were confusingly similar to real rhino horn in look, feel and properties. Spectral and thermal FT-IR, DSC and TGA analysis demonstrated the similar chemical composition and thermo-mechanical properties between the natural and the faux horns.

## Introduction

The horn of the rhinoceros (Rhinocerotidae) is not a horn in the traditional sense like the horn of a cow or the nail of a hoof although it does share some properties^1^. Actually, the rhino’s horn is a tuft of hair growing, tightly packed and glued together by exudates from the sebaceous glands, on the nose of the animal^2^. Native rhinoceros horn has been examined in a several key research papers. Of specific importance for our study are Ryder *et al*.^1^ who clarify the tubular structure of the keratin hair filaments, Hieronymus *et al*.^3^ who examine histological sections of horn tissue by x-ray CT-scanning and light microscopy and Ling^4^ who identified rhinoceros horn comparatively through appearance and microstructure. Other studies examine the amino acid composition of different rhino horns^5^ or the composition of their inorganic elements^6–9^. Table 1 compares the rhinoceros horn with a few functionally i.e. compact resistant animal bio-composites.

**Table 1:** Microstructural features in rhinoceros horn and functionally comparable horn-like bio-materials^15–17^.

Property	Rhino horn	Compact antler	Sheep Horn	Horse hoof
Mid-range tubule density (mm^−2^)	7	36	22	24
Channel diameter (μm)	100	25	100	40
Porosity (%)	6	9	7	3
Work of fracture (MJm^−2^)	10.0	13.9	19.6	12.0

Note that, in effect, the rhino horn is an all keratin structure and the antler is an all bone structure while the sheep horn is a bone core bearing a keratin sheath and the horse hoof is a keratin nail over a bone base.

Rhino survival is critically challenged by the trade in horn, and a range of horn substitutes are being developed and apparently marketed with the ultimate goal of undermining the market in this much sought after if generally banned commodity. Here we offer an economic substitute for rhino-horn fabricated from the tail hair of its near relative, the horse (*Equus ferus caballus*). As substitute for the sebaceous gland protein exudate cement of the rhino we use a silk-protein based organic filler to glue together the bundled hair. The composite is easily moulded into a horn of hair that is surprisingly similar visually in external and internal micro-structure as well as in feel and overall appearance if shaped and polished.

## Results

**Figure 1:**
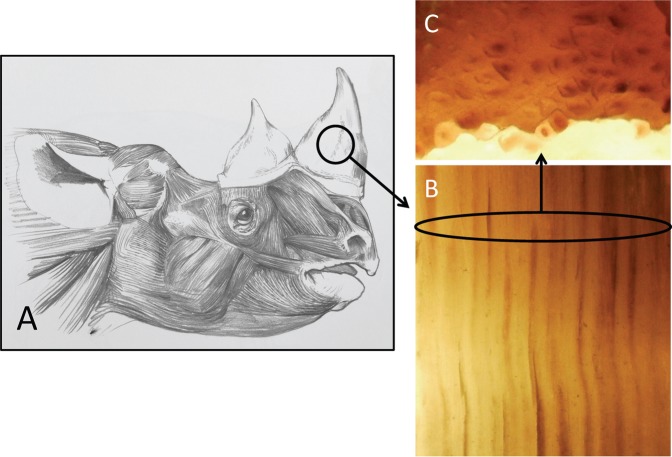
Schematic of Black Rhinoceros (*Diceros bicornis*) horn showing a section of horn with the hair tubules. The rhino head drawing is by Jonathan Kingdon (reproduced with permission). A single hair is circa 200 µm in diameter (length-section **B**, cross-section **C**).

**Figure 2:**
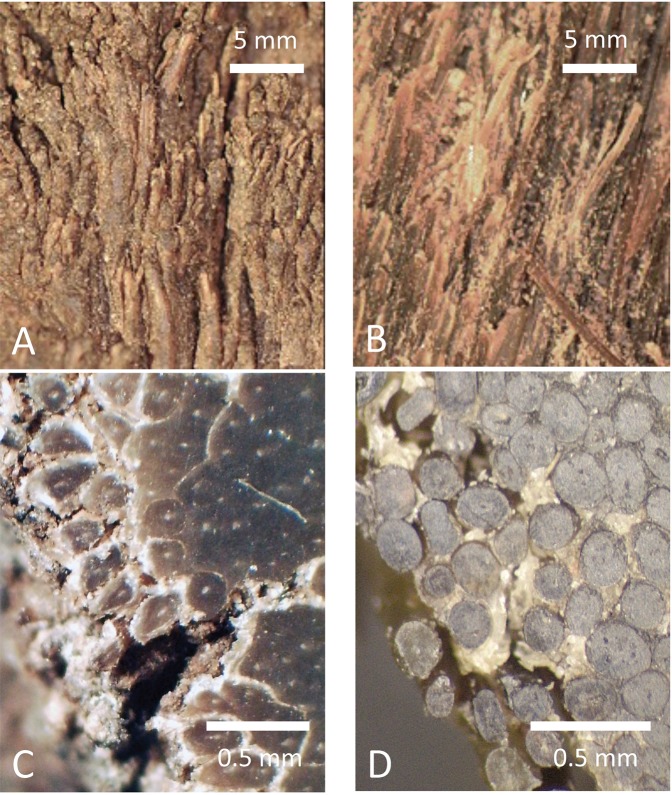
Images of cross-section of a real rhino horn (**A,C**) and an artificial horn (**B,D**). We note that the hair filament density of our artificial rhino horn is about 9 mm^−2^, which is close to that (7 mm^−2^) of real horns^11^.

The horn of the rhino consists of hairs tightly packed in the bulk of the protuberance and more loosely arranged at the outer shell (Fig. 1). The matrix material filling between the hairs is a very dense packing of cornified dead skin keratocyte cells that can be heavily pigmented with melanin^3^. Melanin is an interesting pigment that not only provides black colour but may also add to a material’s structural integrity^10^. Thus the native rhinoceros horn in essence is a composite material, structured by its growth, with the tubules of keratin hair forming ‘fibres’ that are embedded in a matrix material that may change in composition along and/or across the horn^11^. Throughout the rhinoceros horn each hair filament retains much of its natural hair structure including the medullary cavity although it is lacking the outermost layers of scaly cuticle so typical for external hairs^3^ (Figs 1B,C and 2A,C).

**Figure 3:**
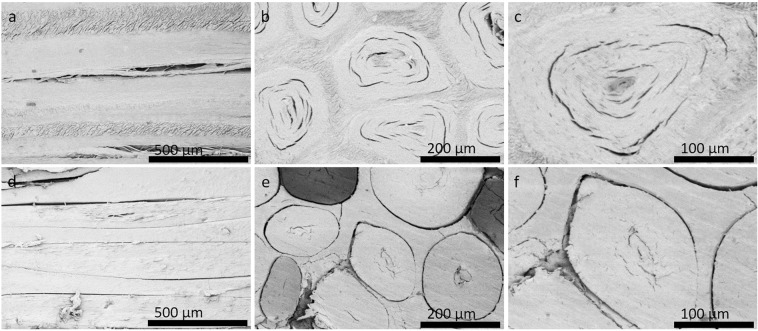
SEM Images of both natural and faux Rhino Hair Horn. The natural horn (upper row) and our faux horn (lower row) show a length section (**a,d**) and two cross sections in two different magnifications (**b,e,c,f**). Note that not all hairs are perfectly circular, while their partial disintegration is probably due to vacuum induced dehydration.

As the key structural material for the manufacture of our artificial rhino horns we used horsetail hair because of its phylogenetic origin (which suggests comparable chemical keratin composition) and its homologous morphological structure (which suggests comparable mechanical properties). Importantly, horsetail hairs also share with rhino horn hairs comparable dimensions, circular symmetry and spongy core structure (Figs 2 and 3). In order to copy the key feature separating the two, the lack of the outermost scaly layers in the rhino horn hairs, we used a Lithium Bromide (LiBr) wash to etch and remove the outer layer of the horsehair. We note that this treatment also facilitated the adhesion between the hair fibres and the matrix material that we used.

As there is no detailed information on the composition of the rhino’s nose-tip exudate and horn matrix material other than that it seems to be a sebatious gland exudate full of deceased highly melanised cells^10^. Such cells would contain high levels of intra-cellular proteins as well as carrying along the rather adhesive extra-cellular fibronectin glycoprotein. Thus the matrix of the native rhino horn would in essence be a largely proteinaceous glue with inclusions of soil and plant sap where the animal has rubbed the growing horn. Assuming such a highly proteinaceous and sticky horn matrix we used for this function in our faux horns the RSF silk fibroin, which we know how to prepare and deploy^12^. Importantly, the RSF material we used can also easily be moulded and cured into a tough matrix to fill-in between the horse-tail hairs.

By bundling the LiBr washed hairs as tightly as possible while infusing them with the RSF solution we were able to create solid composite cylinders of hair-horn. The smaller horn (around 4 cm diameter and 10 cm length) cured within a few days while the largest one (around 12 cm diameter and 35 cm length) took weeks in the vacuum oven to dry. The smaller ones, which were our focus for analysis, filed and polished very nicely into surfaces rather similar, indeed confusingly similar, to surfaces of native rhino horn naturally polished by rubbing. If carefully polished a faux horn could thus be easily modified to resemble the outside of a rhino horn. On the microscopic level our Light and Scanning-Electron Microscopy confirmed that not only the gross morphology and anatomy of the faux horn but also the more detailed fine structure was similar to those of real rhino horn.

Importantly for our more fundamental interests in the novel material, rather than the more superficial copying of structures, was the analysis of its material qualities. To this effect we used DSC and TGA to investigate the similarity of the thermal properties between samples of our artificial horns and the real horn.

Differential Scanning Calorimetry (DSC) is a thermo-analytical technique comparing the heat required to increase the temperature of a sample and a reference allowing us to study physical transformations such as phase transitions and determine whether the process is exothermic or endothermic as well as indicating a glass transition. Thermal Gravimetric Analysis (TGA), on the other hand, measures the mass of a single sample as it changes with temperature over time. This data allows us to probe not only physical phenomena such as phase transitions between the solid, liquid and gaseous states of the various components of the material studied but also chemical phenomena such as thermal decomposition and reactions between surfaces.

**Figure 4:**
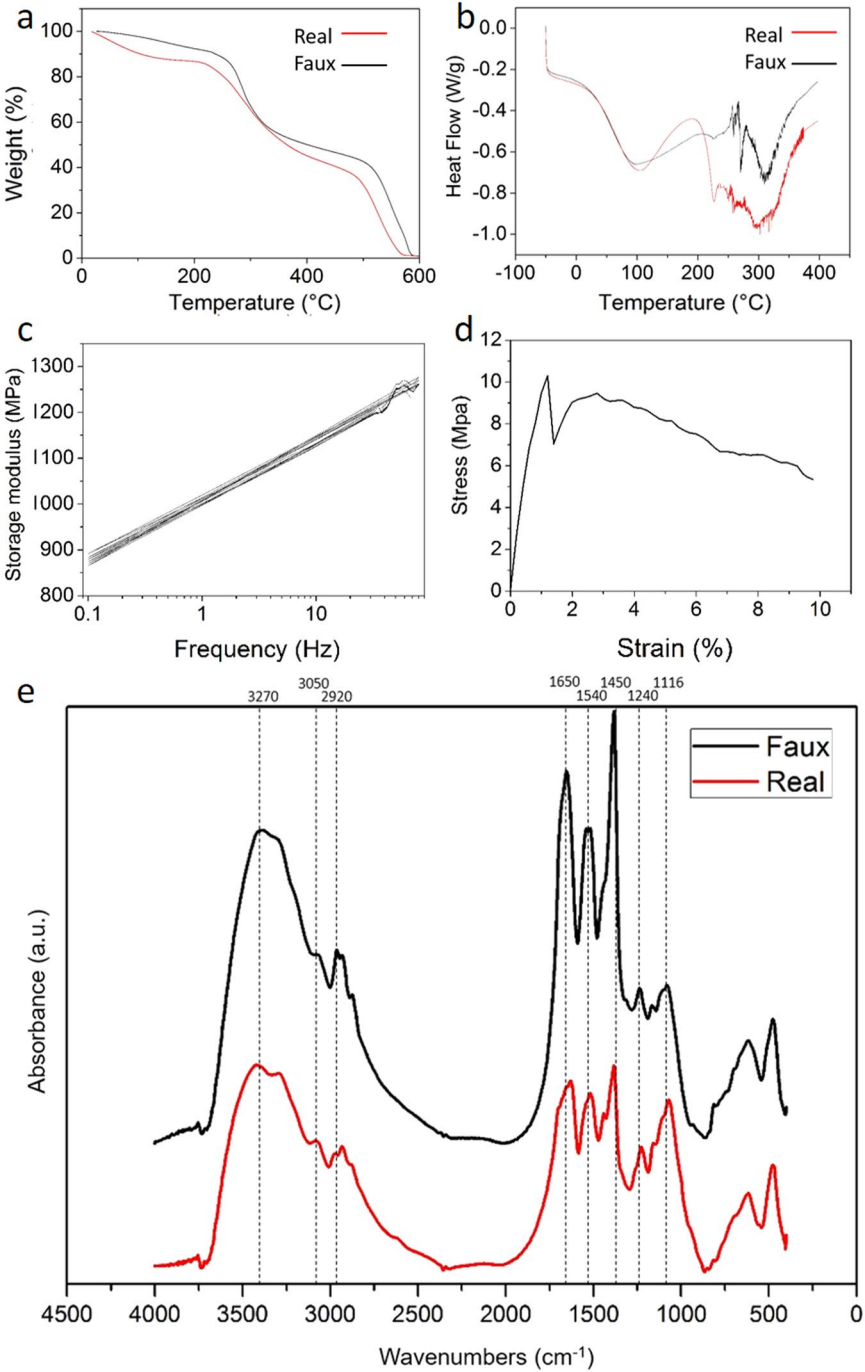
Thermal, spectral and Mechanical testing of the real rhino horns and artificial horn copies. The TGA tests (**a**), the DSC tests (**b)**, the DMTA frequency sweep (**c**) and strain sweep (**d**), the FTIR Spectroscopy result (**e**).

As shown in Fig. 4(a) the DSC analysis demonstrated that both materials were surprisingly similar with that peaks at 100 °C indicating the insipient moisture of the samples while the broad endothermic peak from about 200 °C to 400 °C indicates the degradation of the protein. The data in Fig. 4(b) on the other hand, shows that both real and artificial horns started to decompose approximately at 200 °C with final residues of 1.5 wt% and 1.3 wt%, respectively.

To further probe the chemical composition and properties of both materials we applied the non-destructive method of FT-IR spectroscopy and compared the absorption bands of key constituent molecules of the both artificial and natural horn material^8^. The absorption bands observed (Fig. 4) at 1650 cm^−1^ and 3050 cm^−1^ were assigned to C=O stretching and N-H stretching. 3270 cm^−1^ belongs to O-H stretching vibration; 1540 cm^−1^ belongs to C=C stretching vibration. 1116 cm^−1^ was the S=O asymmetric stretching, while 1040 cm^−1^ was the S=O symmetric vibrations. 1450 cm^−1^ and 1240 cm^−1^ attributed to C-H bending and P=O stretching. Importantly, samples from both real and artificial horns showed very similar infrared spectra.

Perhaps the most interesting, because mechanically most important, measure of material properties was provided by Dynamic Mechanical Thermal Analysis (DMTA). Here a sample is continuously stress-strained and relaxed by tiny amounts to probe the underlying elastic and plastic properties of a material or composite. In the frequency sweep, samples were tested from 100 Hz to 0.1 Hz, and under that range materials were still in the liner region (Fig. 4). Thus, in the strain sweep, we applied the force with the frequency of 1 Hz, and the elastic modulus was 1.3 GPa, which meets the mechanical properties of natural rhino horns^11^. This similarity between rhinoceros horn and high-performance composites is not surprising; both materials are made up of stiff, inflexible fibres embedded into a flexible resin. The fibres break before they bend while the matrix bends before it breaks. The result is a composite that is able to withstand greater loads than either of its parts. When a stress is applied to the material, the matrix inhibits crack propagation and redistributes stress in the direction of the filaments.

## Discussion

It appears from our investigation that it is rather easy as well as cheap to make a bio-inspired horn-like material that mimics the rhino’s extravagantly expensive tuft of nose hair. We leave it to others to attempt to take our technology further and perhaps even go so far as to fool punters into buying it in replacement or indeed in preference to the real, and extremely expensive, rhino horn. Whether flooding the market with confusing horn copies will ultimately lead to saving rhinos roaming in the wild remains to be seen. As material biologists we see few alternatives. It is for conservation economists to examine whether faus horns do work and then to consider whether driving down the value of rhino horn as described could and would protect the real material and its pachyderm carriers in the wild.

Our artificial silk & horsetail rhino horn mimic did rather well in our comparative analyses. Optical and Scanning Electron microscopy showed similar outer appearance and inner structure between real and artificial materials. Thermal analysis of both horns showed comparable thermal stability. FTIR showed very similar infrared spectra, which would make it rather difficult (and with a little tweaking perhaps even impossible) to distinguish the artificial horn from its rhino model using a handheld spectrometer.

Mechanical tests confirmed that our artificial copy had mechanical properties similar to the natural original. Thus all together, our results demonstrated that a rather simple composite of horse tail-hair embedded in a silk fibroin matrix can provide a fine biomimetic replacement for real rhinoceros horn. Indeed, hair-silk composites might well find uses well beyond fooling superstition as bio-inspired materials. After all, the fundamental structure of the rhino horn is a highly evolved and tough fibre reinforced bio-composite where the hair fibres provide great tensile strength while the silk-protein matrix provides great ductility.

Perhaps of more interest to the scientist is our observation that our horn impostors have analogous thermal stability and chemical composition demonstrated by FT-IR and TGA analysis. Last but not least, the artificial rhino horn provided us with a model for an interesting artificial fibre-reinforced composite with mechanical properties comparable to those of the natural original evolved over millennia.

## Methods

### Regenerated silk fibroin (RSF) solution

The silk fibroin solution was obtained by degumming and LiBr dissolving skeins of commercial Bombyx mori silk fibres with the solution then and dialyzed to obtain the silk protein compounds according to standard protocols^13,14^ giving a protein concentration of approximately 4 wt% which was then stored at 4 °C for further use.

### Pre-treatment of horse hair

Natural un-dyed horse tail hair was purchased online. To etch away the outermost dermic-coat layer, the hair was soaked in LiBr – trialling different concentrations and exposure times for best effect in order to produce hair filaments that most resembled the rhino horn hair, which is lacking outer layers.

### Fabrication of silk-based fibre reinforced composites

The pre-treated horse hair was bundled and embedded in the RSF/HPMC solution typically encased in an open Erlenmeyer vial. The soft composite was placed into a vacuum oven to remove the air and heated under 70 °C for 1 hour. This was followed by soaking in ethanol for 12 hours for ripening and drying in ambient conditions for 48 hrs.

### Characterization of the artificial horn composite

*Scanning electron microscopy* (SEM) observation was performed with a TESCAN TS5136 MM at 20 kV of accelerating voltage to image both surface and cross-section after Au-coating for 30 s. Samples were cut by IsoMet slow saw with diamond wafer blade to create smooth surface. *Thermogravimetric Analysis* (TGA) was performed at 10 K min^−1^ on DTG-60H under air gas with flow rate of 40 cm^3^ min^−1^ at a heating rate of 10 °C per minute from 50 °C to 800 °C. *Dynamic Scanning Calorimetry* (DSC) was performed on small slivers of real rhino horn (legally acquired) and our artificial rhino horn on a TA Q2000 instrument, with a heating rate of 5 °C per minute from −50 °C to 250 °C. For *Fourier Transform Infrared (*FTIR) Spectroscopy the samples were powdered and mixed with KBr, then processed into pellets. The FTIR spectra were obtained with a Nicolet 6700 spectrometer (Thermofisher, the USA) in the range of 650–4000 cm^−1^ with a resolution of 2 cm^−1^ for 128 scans. Mechanical Properties were measured using Dynamic Mechanical Thermal Analysis (DMTA) performed on a TA Q800 under DMA multi-frequency strain mode. The constant parameters for all the DMTA tests are as follows: (i) in the frequency sweep, samples were tested from 100 Hz to 0.1 Hz, (ii) in the strain sweep, we applied the force with the frequency of 1 Hz.

### Origin of the native rhino horn samples

The material analysed in detail came from a documented horn of a Black Rhino (*Diceros bicornis*) collected in 1935 in what was then Southern Rhodesia. This was compared with younger horn material coming from a zoo-bred white Rhino (*Ceratotherium simum*) that had died more recently. This material was very similar in both fine-grained dimensions and structure but different in colour, which would require grey rather than black horse tail hairs for impostor horns. The authors confirm that all methods were carried out in accordance with the relevant guidelines and regulations. The authors also confirm that all experimental protocols were approved by the chair of the licensing committee of the Department of Zoology, University of Oxford, where all the samples resided and were all the testing was done.
